# A Lachesis Case

**Published:** 1883-10

**Authors:** S. Seward

**Affiliations:** Syracuse, N. Y.


### A LACHESIS CASE.

S. SEWARD, M. D., SYRACUSE, N. Y.

December 2d, 1882.—I was called to visit Miss W., aged twenty. Bilious temperament, full habit; spinal tenderness in the whole spine, the most so in the neck, below the occiput; headache in occiput and forehead; the spine has been sensitive and painful for over four years from sitting on stool and practicing at piano; worse from any over doing and cold; can't turn head or move it but little without severe pain in spine of neck into brain; pressure by finger on spine of neck sends pain into brain; pressure on dorsal spine sends pain to stomach, and same lame spine; her digestive functions are not much disturbed; she can't bear to be raised up and can't raise herself up in bed, it causes so severe pain in neck and head; constant pain in spine and head; little sleep.

December 2d.—Prescribed Lach.$^{2c}$ in half a glass of soft water, two teaspoonfuls once in four hours for two days.

December 4th.—Some improvement; Sacc. solution four hours for two days; can't sit up or be raised up; can't turn over.

December 6th.—Improves, less sensitive to pressure all along spine to head and less pain in head and spine.

December 9th.—More pain in head and neck. Lach. 40m, two dry doses four hours apart and then Sacc. solution for one week four hours.

December 16th.—Seems cured of all sensitiveness of spine and headache; gets up, and sits up or takes the bed as she pleases; left her on Sacc. a few days longer; also left two doses of Lach. 40m, if from any cause pain or sensitiveness came again, and to be informed if worse.

